# The Ambivalence of Post COVID-19 Vaccination Responses in Humans

**DOI:** 10.3390/biom14101320

**Published:** 2024-10-17

**Authors:** Radha Gopalaswamy, Vivekanandhan Aravindhan, Selvakumar Subbian

**Affiliations:** 1 Directorate of Distance Education, Madurai Kamaraj University, Madurai 625021, India; radhagopalaswamy@gmail.com; 2 Department of Genetics, Dr Arcot Lakshmanasamy Mudaliyar Post Graduate Institute of Basic Medical Sciences (Dr ALM PG IBMS), University of Madras, Taramani, Chennai 600005, India; cvaravindhan@gmail.com; 3 Public Health Research Institute, New Jersey Medical School, Rutgers University, Newark, NJ 07103, USA

**Keywords:** autoimmunity, proinflammatory response, Bell’s palsy, Guillain–Barré syndrome, immune activation, adverse effects, thrombosis, myocarditis, allergy, long COVID

## Abstract

The Coronavirus disease 2019 (COVID-19) pandemic, caused by severe acute respiratory syndrome coronavirus-2 (SARS-CoV-2), has prompted a massive global vaccination campaign, leading to the rapid development and deployment of several vaccines. Various COVID-19 vaccines are under different phases of clinical trials and include the whole virus or its parts like DNA, mRNA, or protein subunits administered directly or through vectors. Beginning in 2020, a few mRNA (Pfizer-BioNTech BNT162b2 and Moderna mRNA-1273) and adenovirus-based (AstraZeneca ChAdOx1-S and the Janssen Ad26.COV2.S) vaccines were recommended by WHO for emergency use before the completion of the phase 3 and 4 trials. These vaccines were mostly administered in two or three doses at a defined frequency between the two doses. While these vaccines, mainly based on viral nucleic acids or protein conferred protection against the progression of SARS-CoV-2 infection into severe COVID-19, and prevented death due to the disease, their use has also been accompanied by a plethora of side effects. Common side effects include localized reactions such as pain at the injection site, as well as systemic reactions like fever, fatigue, and headache. These symptoms are generally mild to moderate and resolve within a few days. However, rare but more serious side effects have been reported, including allergic reactions such as anaphylaxis and, in some cases, myocarditis or pericarditis, particularly in younger males. Ongoing surveillance and research efforts continue to refine the understanding of these adverse effects, providing critical insights into the risk-benefit profile of COVID-19 vaccines. Nonetheless, the overall safety profile supports the continued use of these vaccines in combating the pandemic, with regulatory agencies and health organizations emphasizing the importance of vaccination in preventing COVID-19’s severe outcomes. In this review, we describe different types of COVID-19 vaccines and summarize various adverse effects due to autoimmune and inflammatory response(s) manifesting predominantly as cardiac, hematological, neurological, and psychological dysfunctions. The incidence, clinical presentation, risk factors, diagnosis, and management of different adverse effects and possible mechanisms contributing to these effects are discussed. The review highlights the potential ambivalence of human response post-COVID-19 vaccination and necessitates the need to mitigate the adverse side effects.

## 1. Introduction

The Coronavirus disease 2019 (COVID-19) is primarily an acute disease of the respiratory system caused by severe acute respiratory syndrome coronavirus-2 (SARS-CoV-2) infection in humans. As of July 2024, about 7.1 million deaths were reported due to COVID-19 worldwide [1]. Although COVID-19 originated in Wuhan, China in December 2019, the disease rapidly disseminated into a global pandemic by March 2020 [2,3]. The disease symptoms included fever, cough, and shortness of breath with additional symptoms like loss of appetite and smell, sore throat, myalgia, malaise, and diarrhea. The severity of the disease varied from asymptomatic cases that cleared SARS-CoV-2 effectively before disease onset to those with respiratory distress and shortness of breath, which can lead to death [3,4]. The COVID-19 cases were classified by the WHO as asymptomatic, mild, moderate, severe, and critically ill patients, with the last category typically manifesting with acute respiratory distress syndrome (ARDS) and death [3,4].

The SARS-CoV-2 belongs to the family of Coronaviruses, which are positive-sense, single-stranded RNA (+ssRNA) viruses that are spherical and encapsulated with crown-like, “spike” proteins [5]. The rapidly evolving (in months) SARS-CoV-2 viral genome encodes for spike (S), envelope (E), nucleocapsid (N), and membrane (M) proteins [6,7]. As an airborne pathogen, SARS-CoV-2 primarily infects the human respiratory system, including the nasal passage, trachea, and lungs, and uses the angiotensin-converting enzyme (ACE2) receptor to enter into various types of immune and non-immune cells, including epithelial, endothelial, myeloid and lymphoid cells in these tissues [7,8]. Following the interaction of SARS-CoV-2 with the ACE2 receptor, a type 2 transmembrane serine protease (TMPRSS2), present in the host cells, cleaves the ACE2 and promotes SARS-CoV-2 entry into the host cell [3,9]. Diagnosis of COVID-19 is primarily made by nasopharyngeal swab testing to confirm the presence of viral RNA by RT-PCR. However, computed tomography (CT) of the chest is performed in severe and critical cases to determine the extent of organ damage [10,11]. Quantitative high-resolution CT can identify ground glass opacities, mixed disease, consolidation, and lung morphology towards improved radiological characterization of COVID-19 disease [12,13]. Treatment options for COVID-19 vary widely depending on disease symptoms and severity, and include analgesics, antiviral drug Remdesivir, Paxlovid, and SARS-CoV-2-specific monoclonal antibodies. Paxlovid is an oral antiviral drug while Remdesivir is used parenterally in hospitalized patients with COVID-19 [14]. Monoclonal antibodies neutralizing SARS-CoV-2 namely Bebtelovimab, Bamlanivimab, Etesevimab, Tixagevimab, Cilgavimab, Casirivimab, Imdevimab are used in COVID-19 infections. They are used as prophylaxis in high-risk cases and for treatment in infected patients. They bind to the S protein of SARS-CoV-2 and neutralize their entry into host cells as well as prevent disease progression [15,16,17]. Corticosteroids were administered mainly to immunocompetent individuals with COVID-19 to alleviate pain and tissue damage as well as to help recover from severe ARDS [2,3,18]. Corticosteroids like dexamethasone, prednisone, methyl prednisone and hydrocortisone were used either orally or intravenously and found to be very effective in an appropriate therapeutic window [19,20].

**Figure 1:**
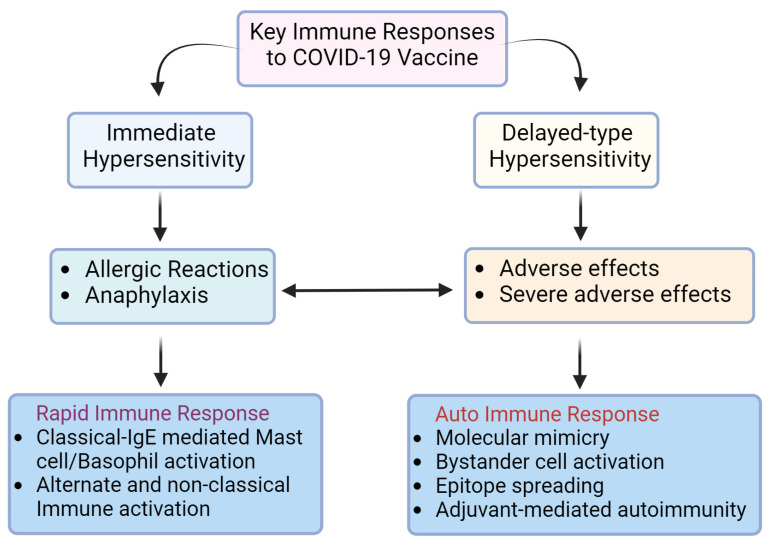
Summary of host response to COVID-19 vaccines. The COVID-19 vaccination-induced host responses can be broadly divided into immediate or delayed hypersensitivity. While the former response elicits allergic reactions and anaphylaxis, the latter response results in mild, moderate, or severe adverse events. The immediate hypersensitivity response is caused either by a classical, IgE-mediated activation of mast cells and basophils or an alternative non-classical pathway involving IgG and other antibodies activating neutrophils and basophils. Autoimmunity due to COVID-19 vaccination can be caused by molecular mimicry, bystander activation of immune cells, viral epitope spreading, or adjuvant-mediated immune response. The overall magnitude and durability of immune response as well as adverse effects mediated by COVID-19 vaccination are determined by several factors, including the age, sex, genetic makeup, immune status, and underlying health conditions of the host as well as the nature of the vaccine used. Image created in Biorender.

The robust and rapid efforts to develop vaccines to protect against COVID-19 resulted in the generation and administration of many different types of vaccines since 2020 [21]. Several strategies, ranging from the application of whole attenuated virus or parts of the virus, nucleic acids, and proteins were used to develop the COVID-19 vaccines. These viral-derived materials were mixed with immunogenic adjuvants, ranging from polyethylene glycol (PEG), CpG motif, lipid nanoparticles (LNP), and alum, and administered to people to protect against COVID-19. The primary objective of all these approaches was to present the immunogenic viral antigen(s) to the host immune cells to trigger a protective immunity against the progression of viral infection into active disease [6,22,23]. The safety and efficacy of all those vaccine types differ among vaccinated individuals, based on the nature of the immunogen and adjuvant, dosage, route of administration, number of doses, and the nature of the individual’s immune system [24,25,26,27]. In general, immunocompetent individuals elicit a quicker and more robust immunogenicity than immunocompromised individuals with underlying health conditions. This disparate immune response significantly affects the host response to SARS-CoV-2 infection as well as the COVID-19 vaccination (Figure 1). Nonetheless, the WHO recommended COVID-19 vaccines for emergency use for patients on an unlicensed basis before the completion of phase 3 and phase 4 trials. Thus, COVID-19 vaccines have helped to significantly reduce mortality due to severe disease and to halt transmission of the infection. Although there is consensus among various stakeholders, including researchers, clinicians, and policymakers on the advantages of COVID-19 on public health, there were also concerns about potential side effects due to the vaccines per se, especially in people with underlying health conditions, which contributes to vaccine hesitancy and compliance with multiple-dose vaccination in the population.

In this literature review, we summarize different types of COVID-19 vaccines and evaluate the adverse effects of the most commonly used vaccines, including their clinical presentation, risk factors, diagnosis, and management. This review is a comprehensive report, indexing the autoimmune and inflammatory response(s) predominantly observed in the cardiac, hematological, neurological, and psychological functions post-COVID-19 vaccinations. We also discuss the various immune mechanisms activated by these vaccines that would have caused different adverse effects in those vulnerable populations. Finally, we commented on the impact of vaccination on long COVID-19 conditions. With various vaccines being used globally, the review focuses only on vaccine types and products reported in the literature for adverse effects, which were mostly the vaccines approved under the emergency use listing.

## 2. Types of COVID-19 Vaccines

The development of COVID-19 vaccines involves multiple approaches that use the virus as a whole; in parts like deoxyribonucleic acid (DNA) or messenger ribonucleic acids (mRNA) or protein subunit antigen(s) in every vaccine type; or delivery using the use of different viral vectors (Table 1). Inactivated viral vaccines contain the virus or the genetic material, do not replicate inside the vaccinated host, and primarily elicit a humoral (antibody) response. Inactivated vaccines can be improved by using adjuvants and are favorable for long-term protection against viral infection. In contrast, SARS-CoV-2 mutant subtypes with low pathogenicity are used as live attenuated vaccines. These weakened virus particles can replicate within the host cells and elicit both humoral and cell-mediated immune responses. Covaxin (Bharat Biotech, Hyderabad, India), Covillo (Sinopharm, Beijing, China), Coronavac (Sinovac, Beijing, China), and COVI-VAC (Codagenix) are typical examples of whole virus vaccines where all except latter (intranasal) are administered intradermally in two doses [6,22,23]. Covaxin (developed by Bharat Biotech), Covilo (developed by Sinopharm, Beijing, China), and Coronavac (developed by Sinovac Life Sciences Co., Ltd., Beijing, China) were approved for use in 14, 93, and 56 countries globally. All three vaccines were listed as emergency use vaccines by WHO in 2021 [28].

The component viral vaccines include protein subunit vaccines, nucleic acid vaccines, viral-like particles, and viral vector vaccines. Due to its inability to display the full antigenic complexity of the virus9, component vaccines are generally considered safe. Protein subunit vaccines are made up of one or more fragments of the virus usually the S-protein that can trigger an immune response and offer moderate immunogenicity against SARS-CoV-2 infection. Protein vaccines are stable and safe, offering moderate immunogenicity usually administered intradermally in two doses. Nuvaxovid (Novovax, Gaithersburg, MD, USA) and COVOVAX (Serum Institute of India, Pune, India) are recommended for emergency use [6,22,23]. Nuvaxovid and COVOVAX were approved for use in 40 and 6 countries, respectively, and listed under emergency use listing by the WHO in December 2021 [28]. A plasmid-based delivery system is used in DNA vaccines that carry a plasmid DNA encoding the viral protein, such as the S protein.

The viral proteins are expressed when they are injected into the host and undergo appropriate species-specific post-translational modifications to trigger an immune response against the infecting virus. DNA vaccines are administered intradermally usually in three doses. Though safe, it offers low immunogenicity and warrants repeated doses. There is only one DNA vaccine, ZyCoV-D (Zydus Cadila, Ahmedabad, India) approved for use in long-term protection but not for emergency use [6,22,23]. The mRNA vaccines (predominantly no-replicating type) deliver the mRNA of the SARS-CoV-2 S protein to be translated into the host cell. The mRNA vaccines can be administered intradermally (ID), intramuscularly (IM), and/or subcutaneously (SC) usually in three doses. Comirnaty BNT162b2 (Pfizer, New York, NY, USA and BioNTech, Mainz, Germany) and mRNA-1273 (Moderna, Cambridge, MA, USA) are commonly used mRNA vaccines worldwide. Though safe, they are unstable and require repeated doses [6,22,23]. BNT162b2 (Pfizer/BioNTech) and mRNA-1273 (Moderna) were approved for use in 149 and 88 countries and listed for use in emergencies by WHO in 2020 and early 2021, respectively [28]. Both the mRNA vaccines encoded the stabilized prefusion S glycoprotein and were recommended for use in a 2-dose schedule [28,32,33]. The multimeric virus-like particles (VLPs) use multimers of viruses and generate humoral, and cell-mediated immunity combined with adjuvants to improve their immunogenicity. Although rapid to manufacture and safe it is not recommended for emergency use. Covifenx is a VLP produced on a plant-based platform and is currently in a clinical trial [6,22,23]. Viral vectors can be replicative or non-replicative where the non-replicative viral vectors (NRVVs) have better safety profiles and replicative viral vectors (RVVs) have greater immunogenicity than NRVVs. Both express the S-protein using the viral vector. ChAdOx1-S (Oxford University, Oxford, UK–AstraZeneca, Cambridge, England) and Ad26.COV2.S (Janssen, Beerse, Belgium) are examples of viral vector vaccines [6,22,23]. The Ad26.COV2.S vaccine that contains an Ad26-based vector and prefusion-stabilized S protein (furin cleavage site mutation and proline substitution) for better immunogenicity was administered in two doses. The ChAdOx1 vaccine contains a ChAdOx1 chimpanzee adenovirus vector expressing SARS-CoV-2 S-glycoprotein.Ad26.COV2.S (Janssen) and ChAdOx1-S (Oxford–AstraZeneca) were approved for use in 113 and 149 countries and listed for use in emergencies by WHO in early 2021 [28,34,35]. Another adenoviral vector vaccine, Sputnik V (Gamaleya Research Institute of Epidemiology and Microbiology, Moscow, Russia) developed at Gamaleya National Research Centre for Epidemiology and Microbiology (Moscow, Russia) uses a heterologous recombinant adenovirus approach with Ad26 and Ad5 as vectors for the expression of the S protein and used in 30 countries [6].

By September 2023, there were reports of 17 million pediatric cases of COVID-19 globally. COVID-19 has the potential to cause severe disease including respiratory distress and multi-organ failure [36]. An estimated total of 1,755,596 (10.1%) children aged 6 months–4 years had received the COVID-19 vaccine as of 31 December 2022. Although data showed that 70% of children from the same age group who received Pfizer-BioNTech or Moderna mRNA-1273 had completed their vaccination series, there is still a low coverage of COVID-19 vaccines in this age group, particularly in the rural area. The vaccine coverage in 2022 for children aged 5–11 years and 12–15 years were 24.0% and 33.3%, respectively. Vaccine hesitancy in children and adolescents is usually due to anxiety and worries about possible side effects [37]. Many of the children and adolescents were homeschooled during the COVID-19 pandemic and parents showed vaccine hesitancy when compared to children who went to public or private schools [38].

Children and adolescents are usually not included in many clinical trials and data on the safety and efficacy of the vaccines on this age group is usually lacking. Analysis of existing information systematically would be beneficial in this situation. COVID-19 during pregnancy poses greater health risks and complications compared to those who are not pregnant. Pregnant women are recommended to take the COVID-19 vaccines to prevent infection. COVID-19 infection during pregnancy contributed to higher rates of SARS-CoV-2-induced intensive care unit (ICU) admission, oxygen supplementations, need for mechanical ventilation, and death [39]. Pregnant women are not included as part of clinical trials and data on the same is lacking. Although it is safe and efficacious to use COVID-19 vaccines during pregnancy, low income and low education contribute to vaccine hesitancy.

Some studies compare the humoral response between naturally infected persons to hybrid immunity where the person is both naturally infected and vaccinated. In all the studies, the study population was tested by RT-PCR for confirmation of the absence of SARS-CoV-2 infection [40,41,42]. The estimate was usually completed as a measure of hazard ratio (HR) or vaccine efficacy (VE), which included patient characteristics and laboratory investigations. Patient characteristics included age, gender, time from previous COVID-19 infection, number of doses of COVID-19 vaccination, and time from last doses with unvaccinated but previously infected persons serving as controls. The laboratory investigations included measurement of neutralizing antibodies against S, N, or receptor binding domain proteins to indicate humoral immune response, and IFN-γ as well as cellular immunity. The studies showed that hybrid immunity conferred better humoral and cellular response as indicated by the levels of neutralizing antibodies and IFN-γ levels [40,41,42]. One study showed that the mean concentration of anti-spike antibody was 3.3-fold higher in hybrid immunity against natural infection. A positive dose–response relationship in comparison with the number of doses received in the case of mRNA and adenoviral vaccines [40]. The hybrid immunity against disease severity was 97.4% and the effectiveness against reinfection was 41.8% following primary vaccination and showed improved protection and effectiveness than natural infection [41]. IFN-Y levels indicated superior T cell immunity in hybrid immunity following mRNA and adenoviral vectors. The increased interval of 13–15 months between infection and vaccination showed positive protection [42]. WHO recommends the integration of infection and hybrid immunity information into immunization strategies and collect more seroprevalence information. Though hybrid immunity is considered superior, it warrants more studies to understand factors contributing to the same and plan better vaccination across different populations [43].

## 3. Adverse Effects of COVID-19 Vaccines

Adverse events (AE) are side effects on an individual after a vaccination. They normally are mild or moderate and resolve within 3 to 4 days. The vaccine-related adverse reactions include fatigue, muscle pain, headache, fever, chills, injection site injury or reaction, sore throat or rash. The onset of mild to moderate side effects was observed within 7 days of vaccination for most vaccine types [44,45]. However, serious adverse events (SAE) are those involving death, life-threatening illness, hospitalization, permanent disability, and birth defects. They are normally identified as part of the clinical trial process and monitored [45,46]. While vaccines are designed to be safe and efficacious, some adverse effects following immunization (AEFI) are usually underreported. It is pertinent to improve the reporting of AEs specifically the ones not detected during premarket testing. The vaccine adverse event reporting system (VAERS) is a web-based vaccine adverse event self-reporting system developed by the Food and Drug Administration (FDA) and Centers for Disease Control and Prevention (CDC) in 1990. The website was created to receive reports about adverse events (AEs) which include medical conditions that occur after vaccination but may not necessarily be caused by the vaccination [47,48,49,50]. Serious adverse events are predominantly observed with mRNA and viral vector-based vaccines; typically affecting males over females; middle-aged over young adults or aged and usually after the first dose (Table 2).

Among children, multi-country studies from seven randomized clinical trials including 10,950 participants from the age group between 6 to 18 were analyzed [51]. mRNA vaccines showed local and systemic involvement including pain at the injection site, headache, and fatigue, but no serious adverse effects. Inactivated viral and DNA vaccines showed similar effects while adenoviral vector vaccines additionally showed fever and gastrointestinal disorders [51].

From different studies of clinical and preclinical trials involving pregnant women using different types of COVID-19 vaccines from different platforms using various adjuvants, results showed no maternal or neonatal adverse events like stillbirth, preterm birth, low birth weight, or lower Apgar scoring [52,53,54,55]. The most commonly reported symptoms in pregnant women with Pfizer-BioNTech BNT162b2 and Moderna mRNA-1273 were pain at the injection site and fatigue with any other adverse events including maternity ones like stillbirth or preterm birth no different from unvaccinated pregnant women [56,57]. These studies reiterate the safety of COVID-19 vaccines in pregnant women. Booster doses are recommended for prolonged humoral and cell-mediated immune responses post-primary COVID-19 vaccinations. Safety, immunogenicity, and reactogenicity of different COVID-19 vaccine types have been studied and shown to be protective recommending up to the fourth booster dose [58,59,60]. A cross-sectional study comprising 106 health workers receiving booster doses of the Moderna vaccine showed mild adverse effects like pain at the injection site, myalgia, fever, and chills [61]. In another systematic review comprising 620,793 individuals taking mRNA vaccines Pfizer-BioNTech and Moderna and adenoviral vector vaccine ChAdOx1 showed local and systemic side effects comprising pain and tenderness at the injection site, myalgia and fever as predominant adverse effects following the booster dose. Further, homologous boosters showed a lower rate of systemic side effects than heterologous boosters [62].

## 4. Immediate Hypersensitivity to COVID-19 Vaccines

**Figure 2:**
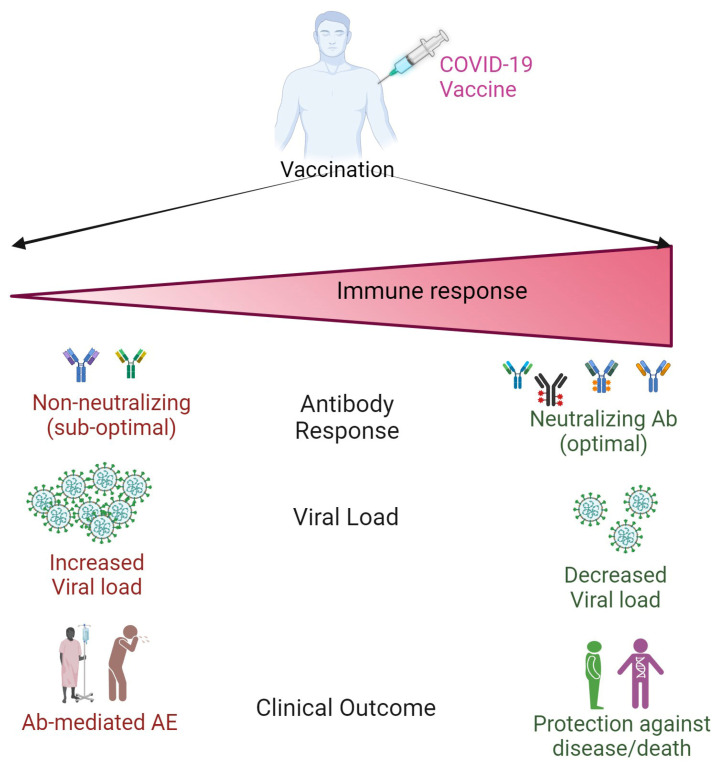
Effects of COVID-19 vaccination-induced immunity. Following vaccination, the immune response against COVID-19 is mediated mainly by the development of Abs against SARS-CoV-2 proteins. The magnitude of immune response developed and its impact on the host protection is determined by the nature of Ab response elicited. An effective neutralizing Ab response neutralizes the virus, controls the infecting viral load and protects the vaccinated host against severe disease and/or death due to infection. However, a sub-optimal non-neutralizing Ab response leads to poor neutralization of the virus and ineffective control of viral load in the organs and may also contribute to Ab-mediated adverse effects (AE), which may enhance the disease manifestations. Image created in Biorender.

The COVID-19 vaccines can elicit both immediate as well as delayed-type hypersensitivity responses (Figure 2). Following vaccination, the immediate hypersensitivity reactions are observed within minutes to hours of vaccination. The symptoms range from simple skin rash to an anaphylactic shock [63]. Immunization stress-related responses cause effects like urticaria, tachycardia, dyspnea, and syncope besides upper respiratory effects like vocal cord dysfunction/inducible laryngeal obstruction. Diagnosis is usually made by measuring blood pressure, oxygen saturation, and clinical presentation. The management is performed by making postural changes, fluid intake, breathing exercises, and intravenous administration of anti-histamines, anti-depressants, or epinephrine where required [64,65]. Typically, anaphylaxis is an immunological reaction, that involves multi-organ failure and essentially comprises symptoms like skin rashes, hypotension, vomiting or abdominal pain, convulsions, bronchospasm, or laryngeal involvement [66,67]. The incidence of anaphylaxis post-COVID-19 vaccination was estimated at 10.67 per million doses. The age of affected people ranged from 10 to 85 years though the maximum cases were reported from 18 to 64 years and females [68,69]. Previous history of allergy, asthma, or allergic rhinitis were predisposing risk factors. The majority (95%) of cases showed recovery was recorded following anaphylactic shock [9,68]. Both the viral S protein component (i.e., mRNA, protein, or peptides) as well as adjuvants such as polyethylene glycol (PEG) are capable of eliciting antibody response in B cells, resulting in antigen- and/or PEG-specific IgE and IgG antibodies (Figure 2).

**Figure 3:**
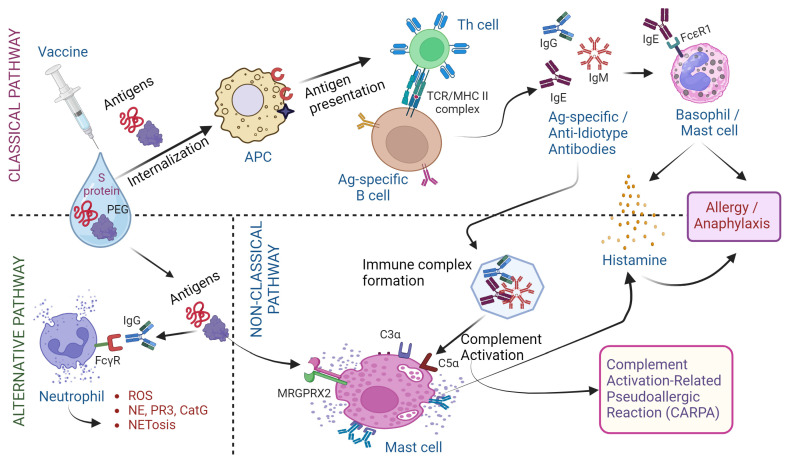
Key pathways of COVID-19 vaccine-induced adverse immune reactions. The COVID-19 vaccine is comprised of the SARS-CoV-2 S protein (either as mRNA or protein) combined with an adjuvant such as polyethylene glycol (PEG). In the classical pathway, internalization of the viral and adjuvant-derived antigens (Ag) in the vaccine by antigen-presenting cells (APC) results in the presentation of antigenic epitopes to the T helper (Th) cells, which produces cytokines and activates Ag-specific B cells to produce various antibodies, such as IgG, IgE, IgM, etc. The Ag-specific IgE Abs binds to the FcεR1 and activates basophils and mast cells to produce histamine, which leads to allergy and/or anaphylaxis reactions. In the non-classical pathway, the antigens were taken up directly by the MRGPRX2 receptor on mast cells, which results in the induction of histamine and allergic responses. In addition, the immune complex formation by the Ag-specific and/or anti-idiotypic IgG, IgE, IgM Abs activates the C3a and C5a complement components, which ultimately results in complement activation-related pseudo-allergic reaction (CARPA). Finally, in the alternative/additional pathway, the antigen–IgG complex is taken up by neutrophils through FcγRs, which activates these polymorphonuclear cells to produce reactive oxygen species (ROS), proteases such as neutrophil-elastases (NE), Protease-3 (PR3), cathepsin G (CatG), and the formation of neutrophil extracellular traps (NETosis). The combined action of these pathways may contribute to the overall allergy and anaphylactic response due to COVID-19 vaccination. Image created in Biorender.

Immediate-type hypersensitivity results in immune responses predominantly in skin, respiratory, and muscular systems with anaphylaxis reported in a few cases. The primary response is caused by IgE or IgG-mediated mechanisms in response to an allergen from the vaccine. PEGylation or the addition of polyethylene glycol (PEG) aims to protect active ingredients from proteolytic enzymes, better solubility, less immunogenicity, and absorption by the body [70,71]. mRNA vaccines against COVID-19 use LNPs as a carrier vehicle to protect the mRNA from degradation and aid intracellular delivery and endosomal escape [72,73]. Moreover, other vaccines like the adenoviral vector vaccine contain polysorbate 80, which contains poly (ethylene oxide) side chains (=CH_2_CH_2_ and =CH_2_CH_2_OH) that share similarities to PEG [74,75]. Previous studies have shown the presence of anti-IgG and/or anti-IgM antibodies in vaccinated healthy individuals [76,77]. One study elucidated the role of anti-PEG antibodies and their role in anaphylaxis in a small population following COVID-19 vaccines. Serum titers for anti-SARS-CoV-2 antibodies as well as PEG IgG and IgM antibodies were measured. This study showed that high levels of anti-PEG antibodies (98% IgG and 99% IgM antibodies) with anti-PEG IgM antibodies were found more predominantly in females than males. Cosmetic use was correlated with higher anti-PEG IgM antibodies in females. Individuals with a previous history of common allergies had higher levels of anti-PEG IgG antibodies. In general, people with hypersensitivity reactions post-COVID-19 vaccination had increased antibodies compared to non-reactors [78]. Following cross-binding of IgE through FccR1 receptors to mast cells or basophils triggers the release of histamines mediating a classical pathway of immediate hypersensitivity reaction. Moreover, the IgG binds to the FcγRs receptor of neutrophils triggering the release of reactive oxygen species (ROS), elastase, and neutrophil extracellular traps resulting in an alternate pathway of immediate hypersensitivity reaction. Another complement-mediated pathway was also identified where allergens trigger complement system C3a and C5a cleavage fragments thereby triggering mast cell degranulation and resulting in CARPA (Figure 3) [74,79]. In mouse models, LNPs could trigger inflammatory responses, characterized by massive neutrophil infiltration, activation of diverse inflammatory pathways, and production of various inflammatory cytokines and chemokines, including the secretion of IL-1β/IL-6 and macrophage inflammatory protein-α and macrophage inflammatory protein-β [80].

In addition to the anti-PEG IgM or IgG antibodies, allergen-specific IgE-mediated anaphylaxis can cause activation of FccR1 receptors on mast cells and basophils. Moreover, the non-IgE-mediated degranulation of mast cells through complement activation-related pseudoallergic reactions (CARPA) results in the generation of C1q, C3a C4, C5a, and Factor B [81,82]. Previous studies reported the presence of anti-PEG and anti-polysorbate IgE and IgG antibodies following COVID-19 vaccination in patients with anaphylaxis and allergy [78,83]. Since naked mRNA in vaccines is highly immunogenic, they are usually encapsulated in LNP formulation to make it less immunogenic. However, under repeated freeze–thaw cycles before the vaccine administration, the mRNA may be released due to disruption of LNP and cause proinflammatory reactions, including anaphylaxis [79,81].

Although all COVID-19 vaccines cause allergic reactions due to immediate hypersensitivity, it is well characterized in mRNA and adenovirus-based vaccines as the adjuvants serve as allergens. Skin prick tests and intradermal tests were completed to characterize the allergen. Polyethylene glycol and tromethamine were observed as potential allergens in mRNA vaccines, the Pfizer-BioNTech BNT162b2 vaccine, and the Moderna mRNA-1273. Polysorbate 80 was identified as an allergen during the usage of adenoviral vaccines, Oxford–AstraZeneca ChAdOx1-S, and Janssen Ad26.COV2.S [68,74].

**Table 2:** Description of various adverse effects post-COVID-19 vaccination.

Clinical Presentation	Incidence/ Prevalence	Predominant Vaccine Type	Age of Onset	Time of Onset	Diagnosis	Treatment	References
**Anaphylaxis** Multi-organ involvement essentially comprises symptoms like hypotension, vomiting or abdominal pain, convulsions, bronchospasm, or laryngeal involvement	10/million vaccinated people	1. Janssen Ad26.COV2. 2. Oxford–AstraZeneca ChAdOx1-S 3. Pfizer-BioNTech BNT162b2 4. Moderna mRNA-1273	18–64 years predominantly	15–30 min	Blood pressure, oxygen saturation, and clinical presentation	Postural changes, fluid intake, breathing exercises, and intravenous administration of anti-histamines, anti-depressants or epinephrine as required	[64,66,67,68,69,71,74,79,82]
**Myocarditis/Pericarditis** Predominantly heart inflammation namely myocarditis, pericarditis, myopericarditis	3–50/million vaccinated people after II dose	1.Pfizer-BioNTech BNT162b2 2. Moderna mRNA-1273 3. Janssen Ad26.COV2. 4. Oxford– AstraZeneca ChAdOx1-S	18–64 years; predominantly reported in 18–29 years	Within 7 days post II dose	Cardiac enzymes (troponin with or without creatinine kinase/CK-MB), inflammatory markers like CRP, ESR or hormones like brain natriuretic peptide, (BNP). CXR; ECG; CT or MRI of the heart; Endomyocardial biopsy (EMB)	Non-inflammatory anti-inflammatory steroids; corticosteroids; and colchicine	[48,50,84,85,86,87,88,89,90]
**Vaccine-Induced Immune Thrombocytopenia** Thrombosis or vaccine-associated thrombosis with thrombocytopenia syndrome (TTS)	0.2–20/million vaccinated people after I dose 2–3/million vaccinated people after II dose	1. Janssen Ad26.COV2. 2. Oxford– AstraZeneca ChAdOx1-S	18–59 years; mean age of 45–48	By 4–42 post I dose	Complete blood count, blood smear, D-dimer and fibrinogen levels, activated partial thromboplastin time, and prothrombin time, and MRI/CT and USG of the chest/abdomen/brain	Anticoagulants, intravenous immune globulin (IVIG), corticosteroids, and targeted therapy drugs	[91,92,93,94,95,96,97,98,99]
**Guillain–Barre Syndrome** Limb weakness (proximal and distal), facial nerve palsy, areflexia, acute inflammatory demyelinating polyneuropathy (AIDP), dysesthesias, decreased deep tendon reflexes, paresthesia	1–10/million vaccinated people after I dose	1. Oxford– AstraZeneca ChAdOx1-S 2. Janssen Ad26.COV2. 3. Pfizer-BioNTech BNT162b2 4. Moderna mRNA-1273	12–86 years; predominantly 50–55 years	1–42 post I dose	CSF analysis; MRI of brain and spine; nerve conduction studies; electromyography	IVIG; plasma exchange; oral steroids; anti-convulsants; interleukin-6 blockers (tocilizumab),	[100,101,102,103,104,105,106,107]
**Miller Fisher Syndrome** Ataxia, ophthalmoplegia, areflexia and diplopia	Rare	1. Pfizer-BioNTech BNT162b2 2. Oxford–AstraZeneca ChAdOx1-S	14–84 years; Median age of 64 years	7–42 days post I or II	CSF analysis; MRI of brain and spine; nerve conduction studies; electromyography	IVIG	[102,108,109,110,111]
**Myasthenia Gravis** Slurred speech, diplopia, binocular diplopia, bilateral ptosis, dysarthria and general muscle weakness	Rare	1. Pfizer-BioNTech BNT162b2 2. Oxford–AstraZeneca ChAdOx1-S 3. Moderna mRNA-1273	13–91 years; Median age of 64 years	1–28 days post I or II dose.	Serum AchR and MuSK antibodies; MRI or CT of the brain; electromyography	Oral or IV dose of Pyridostigmine, plasma exchange, and IVIG	[112,113,114,115,116,117]
**Demyelinating Diseases** Headache, low-grade fever, chills, limb pain, muscle weakness, diplopia, paresthesia, paraparesis, blurry vision, and progressive quadriparesis	Rare	1. Pfizer-BioNTech BNT162b2 2. Oxford–AstraZeneca ChAdOx1-S 3. Moderna mRNA-1273 4. Sinopharm Covilo	19–78 years Median age of 40 years	8–35 days post I, II or III dose	CSF for protein levels, antibody detection in serum or CSF for OCBs, NMO MOG antibodies; MRI of the brain or spine and evoke potential estimation	IVMP, IVIG, plasma exchange, and oral corticosteroids	[118,119,120,121,122,123]
**Bell’s Palsy** Facial weakness, dysarthria, lagophthalmos, dysgeusia, dribbling, drooling and overall facial paralysis	Rare	1. Pfizer-BioNTech BNT162b2 2. Oxford–AstraZeneca ChAdOx1-S 3. Moderna mRNA-1273 4. Sinovac Coronavac 5. Gameleya Sputnik	20–79 years Median age of 49 years	1–42 days post I or II dose, mostly I dose	CT and MRI of the brain, electromyography and nerve conduction studies	Oral or IV corticosteroids including prednisone or methylprednisone, antivirals, IVIG, and plasma exchange	[81,124,125,126]
**Autoimmune Encephalitis** Headache, altered mental status, aphasia, behavioral changes, confusion, loss of attention and concentration, dysarthria, and seizures	Rare	1. Oxford–AstraZeneca ChAdOx1-S 2. Pfizer-BioNTech BNT162b2 3. Moderna mRNA-1273	12–82 years; Median age of 40 years	1–30 days post I or II dose	CSF analysis for pleocytosis and elevated protein levels; CT and MRI of the brain	Methylprednisone—oral or IV	[127,128,129,130,131,132]
**Small Fiber Neuropathy** Weakness, gait disturbances, dysesthesia, palpitations, paresthesia, and dysphagia	Rare	1. Pfizer-BioNTech BNT162b2 2. Moderna mRNA-1273	22–66 years	1–10 days post I or II dose (mostly)	Quantitative sensory testing and nerve conduction studies	IVIG and steroids	[103,115,133,134,135,136]
**Psychotic Illness** Depression, sleeplessness, anxiety, increased psychomotor activity, hallucinations, agitation, irritability, delusion and suicidal attempt	Extremely rare	1.Pfizer-BioNTech BNT162b2 2. Moderna mRNA-1273 3. Janssen Ad26.COV2. 4. Oxford– AstraZeneca ChAdOx1-S	15–57 years Median age of 36 years	0–14 days post I or II dose	Blood and urine profiling; CT and MRI of brain	Antipsychotics, Benzodiazepines, Antidepressants, Methylprednisone, and mood stabilizers	[137,138,139,140]

## 5. Autoimmune and Inflammatory Responses to COVID-19 Vaccines

The WHO’s definition of a post-vaccine adverse event is as follows: “any untoward medical occurrence that follows immunization, which does not necessarily have a causal relationship with the usage of the vaccine”. According to this definition, a minority of individuals may develop adverse effects, including autoimmune syndromes, after vaccination [141]. The immediate and delayed-type hypersensitivity (DTH) reactions to COVID-19 vaccines follow different pathways (Figure 3).

The concept of vaccination and autoimmunity has been much debated for vaccines against tetanus toxoid, hepatitis B. influenza virus, poliovirus, and measles [142,143]. The autoimmune reactions are associated rarely with adverse events as is observed with recent COVID-19 vaccination. The autoimmunity and associated inflammatory reactions leading to AEs are multifactorial—either antigen-specific or antigen-independent events [144,145].

### 5.1. Mechanisms of COVID-19 Vaccine-Induced Adverse Immune Reactions

The main mechanisms through which the COVID-19 vaccine triggers autoimmunity include molecular mimicry, the production of particular autoantibodies, and the role of certain vaccine adjuvants. According to this hypothesis, the adjuvated antigens show some structural similarities with self-antigens thereby inducing an autoimmune reaction. Another mechanism is the activation of “innocent bystanders,” leading to autoreactive T cells, polyclonal activation, and epitope spreading of B cells. However, the pathogenic mechanisms behind the correlation between vaccines and autoimmune diseases are not yet fully elucidated. The first systematic review of the literature on new-onset autoimmune syndromes after the COVID-19 vaccine was published in December 2021 [93]. In this study, 276 published cases were identified. The mainstream cases corresponded to Guillain–Barré syndrome (151 patients) followed by vaccine-induced thrombotic thrombocytopenia (93 cases). Less frequent cases, such as autoimmune liver diseases (eight cases), immune thrombocytopenic purpura (seven cases), IgA nephropathy (five), autoimmune polyarthritis (two), rheumatoid arthritis (two), Graves’ disease (four), or systemic lupus erythematosus (three), have been reported as well.

**Figure 4:**
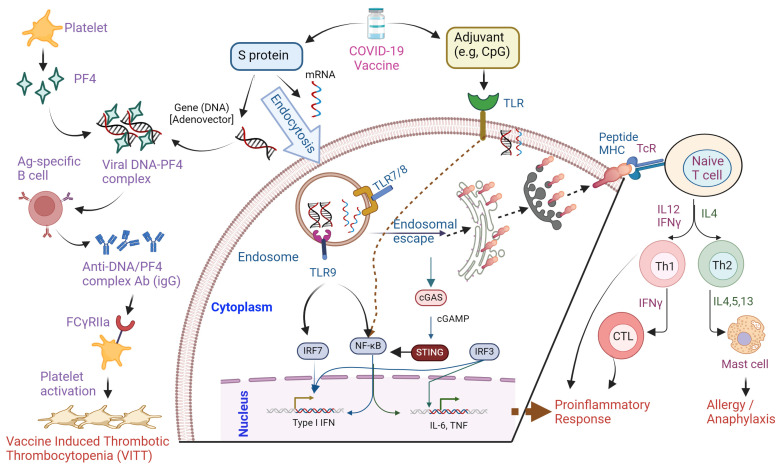
Various mechanisms of adverse immune activation by COVID-19 vaccines. The viral S protein, either as mRNA or recombinant, adenovector-DNA, is endocytosed through Toll-like receptors (TLR) present on antigen-presenting cells (APCs). These endosomes trigger intracellular signaling pathways that result in the activation of Interferon regulatory factor-7 (IRF-7) and nuclear factor k B (NFkB) networks. Activated IRF7 and NFkB upregulate the production of proinflammatory cytokines IL-6 and TNFα. Alternatively, the viral components can escape from the endosome and trigger the cGAS signaling pathway, which activates STING/IRF3 network that ultimately results in the upregulation of proinflammatory type I interferons (IFN) response. Finally, the viral nucleic acids are translated into peptides and presented by the APC to activate T cells through the T cell receptor (TcR). Activation of naïve T cells results in the production of cytokines. Exposure to IL-4 skews the naïve T cells to an anti-inflammatory, Th2-type T cells that produce IL-3, IL-5, and IL-9, all of which can activate mast cells to elicit an allergic/anaphylactic reaction. In contrast, exposure to IL-12 and IFNγ polarizes the naïve T cells into Th1-type cells, which contributes to the proinflammatory response. Apart from the viral-derived molecules, vaccine adjuvants, such as CpG, can be recognized by TLR on the APC, with further activation of the NFkB pathway, leading to the production of inflammatory response. The viral nucleic acids also form a complex with platelet factor-4 (PF4) produced by the blood platelets. This complex activates Ag-specific B cells to produce anti-DNA/PF4 complex IgG, which binds with the FCγRIIa receptor on the platelets and activates these cells to form aggregates, leading to vaccine-induced thrombotic thrombocytopenia (VITT). Thus, both APCs and platelets play divergent roles in mounting immune dysregulation upon exposure to viral antigens and/or adjuvants. Image created in Biorender.

Multiple mechanisms, ranging from activation of proinflammatory and antigen-specific Ab responses to molecular mimicry have been attributed as potential causes for COVID-19 vaccine-induced adverse effects (Figure 4). Molecular mimicry is an autoimmune mechanism where the S protein of SARS-CoV-2 cross-reacts with human antigens and induces immunity. This happens due to cross-reactivity between S protein and self-antigens resulting in antigen presentation by antigen-presenting cells through major histocompatibility class II (MHC class II) to CD4+ T cells [146,147]. Moreover, mRNA vaccines can bind to pattern recognition receptors before their translation and initiate recognition by toll-like receptor (TLR) 3, 7, and 8 inside the endosomes. Alternately, the recognition happens within the cytosol through retinoic acid-inducible gene-I (RIG-I) and melanoma differentiation-associated protein 5 (MDAP5). Both events lead to the induction of proinflammatory cytokines including the assembly of inflammasome pathways with the involvement of Type-I interferons and NF-κB responses [148]. Another mechanism involves the receptor-independent activation of T and B cells by cytokines. Bystander CD8+ T cells are activated primarily by IL-15 association with its specific receptor-α (IL-15R-α) on the surface of the APC. The IL-15 levels increase in response to cytokines IFN-α/β as well as IL-12, and IL-18, which induce IFN-γ. IL-2 is a potent activator of bystander CD4+ T cells [146,149]. Moreover, adjuvants can trigger the TLRs and lead to NLRP3 inflammasome activation in dendritic cells causing innate immunity as well as B or T cell activation, resulting in the onset of adaptive immunity [149,150].

Another mechanism is epitope spreading, in which any tissue damage can lead to the emergence of autoreactive T or B cells against neoepitopes with altered avidity and affinity. Typically, this could be a possible mechanism behind myocarditis and associated cardiac damage. Previous SARS-CoV-2 infection predisposes a person to myocarditis, where the primed T cells will cross-react with cardiac antigens, leading to cardiac cell death [150,151]. There are other candidate B-lymphocyte stimulators, such as anti-S protein antibodies developed after exposure to SARS-CoV-2, that stimulate the production of anti-idiotype antibodies after the anti-COVID-19 vaccine. In addition to targeting self-antigens, these COVID-19 autoantibodies can restructure themselves to display novel antigen epitopes and operate as self-antigens. The antibodies are generated against the viral S protein that binds to the ACE2 receptor in the human respiratory cells. Although these antibodies bind to S, the antibody binding regions can elicit antibody responses called anti-idiotype antibodies or Ab2. They can bind ACE2 and trigger TLRs and cytokine responses. Ab2 antibodies are observed in patients with myocarditis [86,150,152].

Apart from the immune response, secondary infections post-vaccination could occur. Studies conducted previously with SARS-CoV and MERS-CoV have shown the exaggeration of viral infection in vaccinated individuals due to a phenomenon called antibody-dependent enhancement (ADE). The same was observed with COVID-19 with the involvement of IgG antibodies and Fcγ receptor of immune cells [153,154]. Fcγ of immune cells can connect to antibodies and viral proteins, thus initiating an improved viral entry. While the Fab region of IgG antibodies binds to viral epitopes/antigens, it prevents entry into the host. Alternately, FcγR II mediated uptake of the virus into macrophages/monocytes, B cells, or APCs leads to viral invasion and enhanced inflammatory cytokine/chemokine involvement, leading to enhanced disease [153,154,155].

Multicenter studies have been carried out to continue informing the scientific community, in addition to informing on the indisputable advantages of vaccines against COVID-19 in groups of patients who presented autoimmune syndromes shortly after applying the first or second dose of the vaccine. These studies suggest an association between an “adjuvant” of these vaccines and the development of such autoimmune syndromes in genetically and immunologically predisposed individuals. In this context, the participation of age-associated B cells (ABC) in the immune response triggered by the SARS-CoV-2 vaccine has been proposed. These ABC cells (CD11c + T-bet +), or double negative (DN) in humans, expand with age in healthy individuals and are increased early in autoimmune diseases such as SLE and infections. The cells are characterized by generating immunoglobulin G, increasing antigen presentation to T cells, and germinal center formation. Another characteristic of these ABC cells lies in their role in triggering a hyperresponse when stimulated with Toll-like receptors 7 (TLR7) signaling, capable of generating autoreactive antibody-secreting plasmablasts. mRNA/DNA SARS-CoV-2 vaccines use TLR7/8 and TLR9 agonists as “adjuvants,” which may stimulate the subgroup of ABC to form autoantibodies, which can lead to post-vaccine autoimmune syndromes [156,157]. The activation of TLR7 and TLR9 can lead to the production of interferon I, an essential cytokine for the development of SLE and other ARD. There are other candidate B-lymphocyte stimulators, such as anti-S protein antibodies developed after exposure to SARS-CoV-2, which stimulate the production of anti-idiotype antibodies after the COVID-19 vaccination [158]. Lipid nanoparticles or other components of the vaccine have been proposed for vaccine-induced effects [159]. However, the exact mechanisms behind post-COVID-19 vaccine autoimmune syndromes remain to be established.

The respiratory system is the first organ system invaded by SARS-CoV-2. It might also be the first system to be affected by the cross-reaction between the immune response after SARS-CoV-2 infection and pulmonary surfactant proteins since the SARS-CoV-2 S protein and lung surfactant proteins share 13 of 24 pentapeptides. In addition, the cross-reaction between SARS-CoV-2 proteins and a variety of tissue antigens could lead to autoimmunity against connective tissue and the cardiovascular, gastrointestinal, and nervous systems. Infections act as environmental triggers to cause autoimmune diseases triggered by vaccines, while microbial antigens can elicit cross-reactive immune responses against self-antigens. The immune cross-reactivity triggered by the similarity between certain vaccine components and specific human proteins could render the immune system against pathogenic antigens to attack similar proteins in susceptible populations and lead to autoimmune diseases, a process known as molecular mimicry [160,161,162]. Influenza, hepatitis B, and human papillomavirus vaccines have been suspected to trigger autoimmunity through molecular mimicry. In addition, only a minority of vaccinated subjects subsequently developed autoimmune phenomena, indicating a genetic predisposition to vaccine-induced autoimmunity.

Vaccines trigger the adaptive immune response to confer protection against disease progression, which may also stimulate a hyperinflammatory condition. Post-vaccination healthy individuals exhibit acute increases in type-I IFN expression, oxidative stress, and DNA damage accumulation in blood mononuclear cells, coupled with effective anti-SARS-CoV-2-neutralizing antibody production. Sprent and King deem that the side effects of COVID-19 vaccines are simply a by-product of a transient burst of IFN-I generation concomitant with the induction of an effective immune response. However, the production of particular autoantibodies may be responsible for these adverse events. Vaccine-induced thrombotic thrombocytopenia (VITT) events have been widely reported, which can plausibly attributed to platelet factor 4 (PF4) antibody-mediated platelet activation through IgG-FcγR interactions [163]. Also, complement activation triggered by anti-PF4 antibodies appears to be implicated in VITT. However, Greinacher et al. found that PF4 antibodies induced by vaccination do not cross-react with the SARS-CoV-2 S protein [164].

Vaccine adjuvants could render vaccine immunogenicity by triggering the NLR pyrin domain containing 3 (NLRP3) inflammasomes. The mRNA contained in mRNA vaccine presents as both antigen and adjuvant and is identified by endosomal Toll-like receptors (TLRs) and cytosolic inflammasome components, triggering inflammation and immunity [165,166]. The NLRP3 inflammasome displays a vital role in the innate and adaptive immune system, as well as its contribution to several autoimmune diseases, including rheumatoid arthritis, systemic lupus erythematosus (SLE), Sjögren’s syndrome, systemic sclerosis, and ankylosing spondylitis [165,166].

### 5.2. Direct Toxicity of COVID-19 Vaccine Components

The S protein is the most important active ingredient of almost all the COVID-19 vaccines manufactured to date. This is basically because of the following reasons: 1. Being an immunodominant antigen, high titer antibodies can be easily generated against this antigen; 2. Being a surface antigen, neutralization of this protein can actively inhibit viral entry, which is the most crucial step in viral replication; 3. Antibody-mediated neutralization of the viral particles will not only make them ineffective but will also opsonize them, making them more vulnerable to phagocytosis by macrophages. In spite of these advantages, high circulating levels of S protein (which is often seen after vaccination) can cause systemic and organ-specific toxicity. ACE2 and TMPRSS2 are the host receptors for the S protein. These receptors are widely expressed in several cell types and account for the systemic as well as organ-specific toxicity of S protein. ACE2 is a vital element of the renin–angiotensin system (RAS) and is primarily found in epithelial cells of various organs, including the heart, small intestine, kidney, testis, trachea, bronchi, alveoli, respiratory tract monocytes, and alveolar macrophages. The RAS comprises two arms: one that produces angiotensin II (the active hormone) from angiotensinogen and another that breaks down Ang II through ACE2. ACE2 is a counter-regulatory mechanism for RAS. ACE2 expression is elevated under conditions of COPD (chronic obstructive pulmonary disease), smoking, air pollution [6], cardiovascular diseases, and diabetes. Aging is another factor associated with ACE2 hyperexpression. Whether these subjects suffer due to vaccine-induced chronic side effects would be of major interest in the coming years. TMPRSS2, the second receptor for S protein, is highly expressed in multiple organs and plays a significant role in viral entry. It exhibits high expression in various organs, such as the breast, kidney, pancreas, bile duct, prostate epithelial cells, stomach, ovary, small intestine, lung, salivary glands, and colon [167]. Recently, direct binding and activation of TLR2/TLR4 on CD56bright NK cells was reported for the S protein, which can account for the chronic inflammation seen in some vaccinated subjects [168]. Other components of COVID-19 vaccines, such as lipid nanoparticles, are also involved in possible cytotoxic effects. The common thread connecting these adverse events is endotheliopathy due to glycocalyx degradation. This is caused by the depletion of glutathione and inorganic sulfates, shear stress from circulating nanoparticles, lipoprotein aggregation, and the formation of coronas, leading to imbalanced immune responses, ultimately resulting in oxidative stress and systemic inflammation [169].

## 6. Cardiac Manifestations

After receiving COVID-19 vaccines recommended for emergency use, millions of people worldwide reported cardiac dysfunction, predominantly inflammation of the heart, myocarditis, pericarditis, and/or myopericarditis as adverse events. Importantly, myocarditis and pericarditis were reported as adverse events for many other vaccines like smallpox, anthrax, influenza, hepatitis B, typhoid, and varicella virus, though at a much lower frequency compared to the COVID-19 vaccines [170]. Myocarditis involves inflammation of the heart muscle, while pericarditis refers to inflammation of the lining outside the heart; and when both conditions occur together, it is termed myopericarditis. Studies have shown that the severity of myocarditis and pericarditis varies with age, sex, the interval between doses, and the vaccine administered post-COVID-19 doses.

Some of the mechanisms involved in the development of myocarditis after COVID-19 vaccination include the engagement of ACE2 receptors of cardiomyocytes by the viral S protein from the vaccine, which induces inflammation and cellular damage [81,171]. The T cells primed by a previous SARS-CoV-2 infection can react to the S protein after vaccination and attack the cardiac myocytes [81,84]. Similarly, the molecular mimicry between the S protein and myocardial α-myosin heavy chain can cause cross-reactivity and myocardial damage [81,171]. In addition, the heart-reactive autoantibodies, including IgG and IgM, have been identified in patients with myocarditis following COVID-19 vaccination. However, it is debated whether this is pathogenic in itself or the effect of myocardial inflammation [81,85,172].

***Incidence and prevalence:*** The overall incidence of myocarditis and pericarditis collectively across all age groups ranges from 0.3–5.0 per 100,000 individuals [88]. A previous study on the worldwide distribution of myocarditis adverse events following COVID-19 vaccination (36 million doses) was completed using 37 case reports, 27 case series, 6 cohorts, and 2 cross-sectional studies. This study showed that the mRNA vaccine has a higher incidence rate than the non-replicating adenoviral vaccine. The analysis showed Moderna mRNA-1273 had an incidence rate of 40 per million vaccinated individuals, followed by Pfizer BioNTech with 22 per million vaccinated individuals. Both Astra Zeneca ChAdOx1.S and Janssen Ad26.COV2.S had an incidence rate of 15 per million vaccinated individuals [90]. The prevalence of myocarditis and pericarditis among age groups 18–64 and the predominantly male population was reported for all vaccine types [90,170,173,174,175]. The adverse event was reported predominantly after the second vaccination dose, usually given after 3 weeks and up to 7 weeks after the first dose. The median time to onset of symptoms after administration of the vaccine was found to be less than 7 days [48,89,90,176].

***Clinical Presentation:*** The clinical presentation for cardiac dysfunction showed a spectrum of symptoms, including asymptomatic ones like headache, fever, fatigue, and malaise, as well as cardiac symptoms like chest pain, dizziness, dyspnea, effort intolerance, and diaphoresis [49,88,90,173,174,175,177]. However, studies have shown that chest pain, dyspnea, and fever were the most predominant symptoms observed in myocarditis, pericarditis, and/or myopericarditis [87,170,177].

***Diagnosis:*** The signs of cardiac dysfunction are usually identified through biomarkers and laboratory diagnosis. The diagnosis of COVID-19 vaccination-induced cardiac dysfunction is similar to a routine diagnosis of myocarditis or pericarditis that includes clinical findings, laboratory findings, and imaging results. The clinical findings, which are predominantly cardiac symptoms, prompt laboratory diagnosis, causing a bias in identified cases. Myocarditis/pericarditis biomarkers include cardiac enzymes (troponin with or without creatinine kinase/CK-MB), inflammatory markers like C-reactive protein (CRP) and erythrocyte sedimentation rate (ESR) or hormones like brain natriuretic peptide, (BNP). Troponin levels were elevated 48–72 h after symptom onset. The inflammatory markers like CRP and ESR, as well as BNP, were also elevated in myocarditis and pericarditis [47,88,173,177,178]. Imaging procedures include electrocardiogram, echocardiogram, chest radiograph (CXR), chest computed tomography (CCT), and cardiac magnetic resonance imaging (CMR) for post-COVID-19 vaccination-induced myocarditis and pericarditis. Chest radiography (CXR) findings are usually within normal limits, with some reported abnormalities, including pulmonary edema, cardiomegaly, congestion, and pleural effusion [47,179,180]. Echocardiogram findings include increased ventricular wall thickness, decreased segmental contractility, particularly in the inferior and inferolateral walls, increased myocardial echogenicity, and pericardial effusion. ECG changes are most commonly non-specific and subtle, such as mild diffuse ST-segment changes, PQ segment depressions, or non-specific ST-segment changes, similar to classic myocarditis or myopericarditis [88,173]. CCT or CMR is very useful for the definitive diagnosis of myocarditis to detect inflammation as well as measure left ventricular ejection fraction (LVEF). Endomyocardial biopsy (EMB) is another gold-standard diagnostic method for detecting myocardial inflammation [88,173,181,182].

***Treatment and management:*** Non-inflammatory anti-inflammatory steroids and colchicine offer supportive therapy for COVID-19 vaccination-induced myocarditis and pericarditis [174,175,182]. Short-term corticosteroids in patients with severe LV dysfunction or patients presenting with cardiogenic shock are recommended [88,174,177]. The treatment generally for LVEF or heart failure medications includes routine angiotensin receptor–neprilysin inhibitors or angiotensin-converting enzyme inhibitors, beta-blockers, mineralocorticoid receptor antagonists, and sodium–glucose cotransporter-2 inhibitors. In severe LVEF cases, mechanical circulatory support and/or extracorporeal membrane oxygenation (class IIa) in left ventricular dysfunction to unload the left ventricle. If an arrhythmia is present, management should follow the guidelines for arrhythmia [88,173,177].

## 7. Hematological Manifestations

Viral infections rarely also cause thrombocytopenia apart from respiratory symptoms and are reported in a variety of SARS-CoV infections, including SARS-CoV-2. There is a remarkable reduction in platelet count due to various immune-mediated mechanisms [92,183,184]. The vaccine-induced immune thrombocytopenia and thrombosis (VITT) is a rare but severe adverse effect of post-COVID-19 vaccination, characterized by thrombocytopenia and major venous or arterial thrombosis. This condition is also known as vaccine-associated thrombosis with thrombocytopenia syndrome (TTS), with extreme activation of platelets and the coagulation system, leading to a high risk of death from venous or arterial thrombosis or secondary hemorrhage. These adverse events were reported predominantly in cases vaccinated with the non-replicating adenoviral vector vaccines, Janssen Ad26.COV2.S and Oxford–AstraZeneca ChAdOx1-S.

***Incidence and Prevalence:*** The incidence of VITT from the adenoviral vaccines ranges from 0.2 to 20 per million individuals after the first dose. Following the second dose, the incidence decreases to about 2–3 per million post-second dose [99,185]. A recent study showed that VITT was reported in about 300 million vaccine doses of either adenoviral vector vaccine type across different countries. The total cases per million reported ranged from 0.4 and 0.23 cases in Brazil and South Africa, 3.7 cases in the U.S, 1.7 cases in Europe, with ChAdOx1-S vaccination causing 17.6 cases in Nordic countries, and 10 cases per million doses in the UK, compared with 0.2 cases per million doses in Asian countries [99]. The mean age of onset ranged from 18 to 49 years, and the mean age of 45 years. The female population showed preponderance in VITT following vaccination [96,186,187,188]. The adverse event was reported predominantly after the first vaccination dose, usually after the first dose, with a median time to onset of symptoms after administration of the vaccine typically between 4 and 42 days [99,185].

***Clinical Presentation:*** The clinical presentation for VITT showed a range of neurologic/hematologic symptoms based on the extent of the adverse effect. Based on case reports and observational studies, classic symptoms usually include headache, myalgia, nausea, fever, vomiting, pain in the neck, leg, or abdomen, blurred vision, hemiparesis, and bruising or petechiae [91,94,185,186,189]. However, based on the type of VITT, the symptoms are more defined. Cerebral sinus venous thrombosis (CSVT) presents itself as persistent and severe headache, nausea, stiffness of the neck, papilledema, double or blurred vision, focal deficits such as mono paresis or hemiparesis, seizures, and stroke [94,95,190,191,192]. Pulmonary embolism or acute coronary syndrome presents itself as shortness of breath or chest pain, splanchnic vein thrombosis, and abdominal pain [191,193,194,195]. Deep vein thrombosis involves the formation of a blood clot in a deep vein that presents as swelling of the limbs, redness, pallor, or coldness and is typically observed in the lower leg, thigh, or pelvis [91,185,191].

***Diagnosis:*** Once the clinical diagnosis is made based on symptoms, the laboratory diagnosis includes laboratory tests like complete blood count, blood smear, D-dimer and fibrinogen levels, activated partial thromboplastin time, and prothrombin time and imaging using magnetic resonance imaging, and ultrasound or computed tomography of the chest/abdomen/brain [164,196]. A platelet count of less than 150,000/mm^3^ or over 50% platelet reduction from the previous count indicates thrombocytopenia. The D-dimer is a by-product of the blood clotting and break-down process, which is measured in blood. If D-dimer is elevated >4000 fibrinogen equivalent units (FEU) and fibrinogen levels decrease to less than 2 g/L, it is all indicative of blood clot disorders. A raised international normalized ratio/prothrombin time (INR/PT) and activated partial thromboplastin time (APTT) can confirm the presence of VITT. Further confirmation is provided by a positive platelet factor 4 (PF4)/heparin (polyanion) antibody tested in ELISA. Based on the panel of these laboratory tests, VITT is considered confirmatory, and an approach to management is planned [91,101,164,185,188,196,197]. Imaging is usually performed based on the location of the symptom. With persistent headache and neurological symptoms, CT or MRI of the brain along with CT venogram is useful for diagnosis of CSVT. Doppler scans and CT or MRI are useful in diagnosing SVT or DVT and screening the abdomen, pelvis, and limbs based on the clinical diagnosis. CT pulmonary angiogram is used for definite diagnosis of pulmonary embolism. MR angiography (MRA) is a useful tool for the assessment of blood flow to the brain, arteries, and other parts of the body [91,95,164,185].

***Treatment and management:*** The pharmacological management of VITT includes a variety of anticoagulants, intravenous immune globulin (IVIG), corticosteroids, and targeted therapy drugs. Primarily, a complete therapeutic dose anticoagulation in consideration of body weight and kidney tolerance remains the primary treatment modality in all VITT cases. Anticoagulants include heparinoids (unfractionated heparin (UFH), low molecular weight heparin (LMWH), and penta saccharides), vitamin K inhibitors, and direct anticoagulants that reduce coat formation. Direct oral anticoagulants (DOACs), such as dabigatran, apixaban, rivaroxaban, edoxaban, warfarin, and fondaparinux; parenteral direct thrombin inhibitors (e.g., bivalirudin and argatroban); indirect anticoagulants (e.g., fondaparinux, danaparoid); direct thrombin inhibitors (e.g., argatroban, lepirudin) are used for management of anticoagulation in VITT [91,185,187,198]. Alternately, IVIG reduces platelet activation and increases the platelet count by competitively inhibiting the interaction of VITT antibodies [91,94,98]. Corticosteroids inhibit immune response, and methylprednisolone, dexamethasone, or prednisone is administered orally or intravenously to treat VITT [185,195,196,198]. Rituximab, an anti-CD20 monoclonal antibody; Eculizumab, a monoclonal antibody directed against complement C5; and Ibrutinib, a Bruton tyrosine kinase inhibitor, have been used in different studies to block platelet activation and management of VITT [97,199,200,201].

## 8. Neurological Manifestations

Recent studies have shown that both SARS-CoV-2 infection and/or COVID-19 vaccination can induce a spectrum of neurological manifestations in the central and peripheral nervous system [103,202]. These studies also suggest the overlapping and sharing of etiopathogenesis between infection and vaccination. However, the discriminating symptoms or mechanism of onset of ailments between these two conditions (i.e., infection versus vaccination) remain unknown. A multitude of factors, including neuroinflammation due to ACE2 receptor engagement of viral S protein and immune cell dysfunction, has been suggested to contribute to the neurological dysfunction during post-COVID vaccination, many of which are also shared by the SARS-CoV-2 infection [93,129,203]. Some of the adverse neurological complications noticed after COVID-19 vaccination, particularly after the administration of the adenoviral vaccines (Oxford–AstraZeneca ChAdOx1-S and Janssen Ad26.COV2), as well as mRNA vaccines (Pfizer-BioNTech BNT162b2 and Moderna mRNA-1273), includes the Guillain–Barré syndrome and its variant Miller Fisher syndrome (MFS) [93,204].

### 8.1. Guillain–Barré Syndrome and Miller Fisher Syndrome

***Incidence and Prevalence:*** The overall incidence of Guillain–Barré syndrome (GBS) occurs from 1–10/million cases post COVID-19 vaccination, with the highest incidence reported for adenoviral vector vaccines, Oxford–AstraZeneca ChAdOx1-S and Janssen Ad26.COV2 [100,105,107,205]. The incidence and prevalence of Miller Fisher syndrome (MFS) are rare when compared to GBS and highest reported with Pfizer-BioNTech BNT162b2 followed by Oxford–AstraZeneca ChAdOx1-S [76,102]. Earlier, GBS was associated as a side effect of vaccines against other infectious diseases like rabies, diphtheria, pertussis, rubella, hepatitis B and A, and influenza [101,104,107,206,207]. Similarly, MFS was observed rarely post-vaccinations with diphtheria–pertussis–tetanus, pneumovax, and influenza vaccine [102]. The causal association between the COVID-19 vaccine and GBS was observed globally. The prevalence was higher in the USA, UK, and India among other countries and higher compared to the other types of vaccines [104,107]. Although GBS was observed between 12 and 86 years, a median age of 50–55 years across different studies and males were the predominant risk groups [104,105,206,207]. MFS is reported between ages 24 and 84 (median age of 64), predominantly males in the case reports observed [102,109]. The time to onset of symptoms was 1–42 days post the first dose with a median time of 13 days [100,101,105,107,206,207]. The time to onset of MFS is 7–42 days, occurring either after post-first or second dose of the COVID-19 vaccine [102,108,109].

***Clinical presentation:*** GBS presents as limb weakness (proximal and distal), facial nerve palsy, areflexia, acute inflammatory demyelinating polyneuropathy (AIDP), dysesthesias, decreased deep tendon reflexes, paresthesia, and rarely respiratory failure [101,104,107]. Predominant clinical symptoms with MFS include ataxia, ophthalmoplegia, areflexia, and diplopia, with case reports indicating the involvement of limb/facial paralysis, paresthesia, and back pain [102,108,109,110,208]. In GBS, molecular mimicry is the predominant mechanism for causing the adverse effects following COVID-19 vaccination. Bioinformatic analyses have shown the cross-reaction between the S protein of SARS-CoV-2 and neuronal proteins, including neural cell adhesion molecule, receptor-type tyrosine-protein phosphatase-zeta, teneurin-4, receptor tyrosine-protein kinase erbB-2, integrin alpha-X, integrin beta-1, attractin, and myelin-associated glycoprotein [106,208]. Moreover, humoral- or T-cell-mediated peripheral nerve ganglioside antibodies can cause nerve damage, leading to GBS [104,209]. Molecular mimicry between viral S protein and host cell gangliosides can lead to the deposition of complement and damage of Schwann cells, causing GBS [104,106,210]. A similar mechanism of molecular mimicry was also observed in MFS with anti-GQ1b IgG antibodies observed following COVID-19 vaccination [102,111].

***Diagnosis:*** Diagnosis includes mild pleocytosis and albumin cytological dissociation in CSF. Moreover, an MRI of the brain and spine is usually completed when limb weakness or palsy is seen. Electromyography and nerve conduction studies have also been completed to assess the level of nerve involvement in GBS and MFS. Moreover, anti-ganglioside antibodies are the most frequent autoimmune marker in all MFS and many GBS cases [104,109,205,207,208].

***Treatment and Management:*** Management of GBS includes IVIG and plasma exchange as the most common method and only former for MFS [101,104,109,208]. Oral steroids were added to IVIG, where it was found beneficial. Anti-convulsants, as well as interleukin-6 blockers (tocilizumab), were added wherever necessary [101,102,104,105,107,108,109,208].

### 8.2. Myasthenia Gravis

Myasthenia gravis (MG) is a rare neurological complication observed after administration of COVID-19 vaccines, predominantly Pfizer-BioNTech BNT162b2 followed by Oxford–AstraZeneca ChAdOx1-S and Moderna mRNA-1273. MG affects people across 13–91 years of age (median age of 64 years), mostly males with onset of symptoms typically 1–28 days post-vaccination of I or II dose [113,114,116,117]. Clinical presentation includes slurred speech, diplopia, binocular diplopia, bilateral ptosis, dysarthria, and general muscle weakness. Diagnosis is usually completed by testing for Acetylcholine receptor (AchR) and muscle-specific receptor tyrosine kinase (MuSK) antibodies in the serum of patients, MRI and CT of brain or affected organs can provide additional diagnostic value. Electromyography shows decrement in repetitive nerve stimulation tests. Treatment includes administration of oral or IV doses of Pyridostigmine, plasma exchange, and IVIG [112,117]. Previous case reports indicate that following COVID-19 vaccination, molecular mimicry between the viral S protein and self-antigens can trigger the onset of MG. In addition, SARS-CoV-2 may cross-react with AchR target proteins on host cells, causing a clonal activation of B-lymphocytes. The activation of auto-reactive T cells as a bystander effect due to the release of previously sequestered self-antigens following COVID-19 vaccination is an alternative mechanism for vaccine-induced MG. Also, adjuvants can trigger an inflammatory response—autoimmune or inflammatory syndrome induced by adjuvants (ASIA) resulting in MG as neurological complications [114,116,117].

### 8.3. Demyelinating Diseases

A demyelinating disease (DMD) is a very rare adverse effect reported following COVID-19 vaccination using Pfizer-BioNTech BNT162b2, Oxford–AstraZeneca ChAdOx1-S, Moderna mRNA-1273, and Sinopharm Covilo vaccines. The age group ranged from 19 to 78 years across different case reports and median age of 40 years, predominantly females. The onset of symptoms was seen between 8 and 35 days post I, II, or III doses of vaccination [118,119,120,122]. The clinical presentation includes acute traverse myelitis (ATM), multiple sclerosis (MS), neuromyelitis optica spectrum disorder (NMOSD), acute disseminated encephalomyelitis (ADEM), and isolated diseases. Symptoms range from headache, low-grade fever, chills, limb pain, muscle weakness, diplopia, paresthesia, paraparesis, blurry vision, and progressive quadriparesis [118,119,120,121]. Diagnosis of DMD included analysis of blood cells/hemogram, CSF for protein levels, antibody detection in serum or CSF for OCBs: oligoclonal bands; neuromyelitis optica (NMO) antibodies and/or myelin oligodendrocyte glycoprotein (MOG) antibodies. MRI of the brain or spine is conducted where required to perform a radiological assessment of nerve damage. Evoke potentials are calculated to study nerve conduction [119,120,121]. Treatment of DMDs included predominantly intravenous methylprednisolone (IVMP), IVIG, plasma exchange, and oral corticosteroids [118,119,120,121]. Natalizumab, Teriflunomide, Rituximab, and Dimethyl fumarate are used for the management of multiple sclerosis [121].

Molecular mimicry between viral S protein and host cell MOG is one of the mechanisms for DMD post-COVID-19 vaccination [118,120,121]). Other mechanisms include the bystander activation of TLR7 and TLR8 in autoreactive lymphocytes and macrophages, which leads to type I interferon production, local immune activation by T and B cell responses, and ultimately DMD in nerve cells [118,120,121]. Overall, MOG-associated pathogenesis is hypothesized to be the result of additional autoreactive T cell activation and epitope spreading. In addition, the immune adjuvants used in the preparation of vaccines can result in adjuvant-dependent ASIA-causing DMD in post-COVID-19 vaccinated individuals [118,120,121,123].

### 8.4. Bell’s Palsy

Bell’s palsy (BP) is a rare neurological complication reported as an adverse event following vaccination with Pfizer-BioNTech BNT162b2, Oxford–AstraZeneca ChAdOx1-S, Moderna mRNA-1273, Sinovac Coronavac, and Gameleya Sputnik COVID-19 vaccines. BP has been indicated as a side effect following influenza viral vaccines earlier but has been reported for COVID-19 vaccines in recent years. The affected people’s ages range from 20 to 79 years, with a median age of 49 years, predominantly females among all vaccine types. The onset of symptoms started predominantly after the first dose, though also observed after the II dose after 1–42 days with a median onset time of 11 days [124,125,211].

The clinical presentation includes facial weakness, dysarthria, lagophthalmos, dysgeusia, dribbling, drooling, and overall facial paralysis [124,211]. Diagnosis includes CT or MRI of the brain, electromyography, and nerve conduction studies to assess the extent of damage [124,125,211]. Treatment includes oral or IV corticosteroids, including prednisone or methyl prednisone, antivirals, IVIG, and plasma exchange [124,125]. Previous case studies have shown that type I interferon production by COVID-19 vaccine components leads to TLRs 3, 7, and 9 mediated immune cell activation and inflammation in BP. Alternately, host immune cell targeting of myelin through molecular mimicry can cause BP post-COVID-19 vaccination [124,126,208].

### 8.5. Autoimmune Encephalitis

Autoimmune encephalitis (AE) is seen as a rare side effect following COVID-19 vaccination, particularly in cases vaccinated with the Oxford–AstraZeneca ChAdOx1-S, Pfizer-BioNTech BNT162b2 and Moderna mRNA-1273 vaccines [127,130,212]. The age group ranges from 12 to 82 years, with a median age of 40 years and predominantly females. The onset of symptoms ranged between 1 and 30 days post-first (in most cases) and second doses [127,130,212]. The clinical presentation includes a range of symptoms like headache, altered mental status, aphasia, behavioral changes, confusion, loss of attention and concentration, dysarthria, and seizures. Diagnosis is usually completed by examination of CSF for elevated protein levels, pleocytosis, CT, and MRI of the brain. Methyl prednisone as an oral or intravenous route is the standard treatment. Additionally, acyclovir, amikacin, ciprofloxacin, cefotaxime, and vancomycin are prescribed to rule out infectious encephalitis. While severe neurological symptoms like seizures are observed, intravenous lorazepam, levetiracetam, phenobarbital, and valproic acid are prescribed [127,128,130,132,212]. Molecular mimicry, bystander host cell activation, and direct activation of immune cells locally due to viral antigens and/or adjuvants are postulated as possible mechanisms for AE in post-COVID-19 vaccinated individuals [130,131,212].

### 8.6. Small Fiber Neuropathy

Recent studies suggest a potential link between peripheral neuropathy, such as small fiber neuropathy, and COVID-19 vaccination or SARS-CoV-2 infection [136,213]. Small fiber neuropathy (SFN) is a rare neurological disorder observed in a few cases after Pfizer-BioNTech BNT162b2 and Moderna mRNA-1273 administration. Patients were predominantly females aged 22–66 years of age with onset of symptoms between 1 and 10 days after I or II dose (mostly) [115,134,135]. The clinical presentation included weakness, gait disturbances, dysesthesia, palpitations, paresthesia, and dysphagia [115,133,208]. Diagnosis is usually completed by quantitative sensory testing and nerve conduction studies to assess the clinical condition. IVIG and steroids are the choices of treatment, along with the alleviation of symptoms using systemic painkillers and local analgesics [115,133,135,208]. Molecular mimicry and bystander immune cell activation have been hypothesized as molecular mechanisms for SFN [115]. However, the involvement of FGFR3 antibodies indicates binding of the RBD of viral S protein with FGFR3 receptors, but with lower affinity than ACE2 receptors, which can cause an immunological response leading to inflammation and an associated SFN post-COVID-19 vaccination [135,214].

### 8.7. Psychological Manifestations

Psychological illness was observed as an extremely rare event observed as a side effect after administration of Pfizer-BioNTech BNT162b2, Moderna mRNA-1273, Janssen Ad26.COV2 and Oxford–AstraZeneca ChAdOx1-S vaccines in sporadic case reports. The age group involved 15–57 years with a median age of 36 years and predominantly females [137,139]. The clinical presentation included depression, sleeplessness, anxiety, increased psychomotor activity, hallucinations, agitation, irritability, delusion, and suicidal thoughts. Diagnosis includes blood and urine profiling, CT, and MRI of the brain wherever required. The treatment includes antipsychotics, benzodiazepines, antidepressants, methyl prednisone, and mood stabilizers to treat psychosis [137,138,139,140]). Although no specific mechanism has been attributed to COVID-19 vaccine-induced psychosis, previous studies using linear and three-dimensional bioinformatics approaches have reported molecular mimicry between SARS-CoV-2 antigens (apart from S protein) and autoimmune CNS proteins [160]. In addition, proinflammatory factors like IL-1, IL-6, and TNF-α, which damage the central nervous system, have been reported in previous case reports of psychosis [140].

## 9. Long COVID and Effect of COVID-19 Vaccination

Multiple research evidence suggests the existence of a new syndrome called long COVID (also called post-COVID syndrome, long-haul COVID, or chronic COVID) characterized by “nonspecific” symptoms and signs such as encephalomyelitis/chronic fatigue syndrome (ME/CFS), including severe fatigue, sleep disorders, cognitive impairments, and different manifestations of autonomic dysfunction. These symptoms start in an individual infected with SARS-CoV-2 at least 3 months after the infection and persist over 2 months or more without any other alternative diagnosis. Long COVID has been reported in almost 10% of COVID-19-infected patients, and over 200 symptoms affecting multiple organ systems have been identified. Fatigue was the most common general symptom, followed by dyspnea or breathing difficulty, loss of smell or taste, and muscle pain, as observed in most studies on long COVID [215,216,217]. Headache, cognitive impairment, and loss of smell or taste were the predominant neurological symptoms, while chest pain, dyspnea, palpitations, and cough were the common cardiopulmonary symptoms. Anxiety, depression, and sleep problems were the most common observations among mental health, while poor appetite and emesis were the common gastrointestinal symptoms. Additional symptoms observed in other systems included blurry vision, eye irritation, tinnitus, dysphagia, dermatological irritations, menstrual problems, blood pressure or circulation issues [216,218,219,220]. Previous studies have shown that females, older age, higher body mass index, history of smoking, diabetes, obesity, cardiovascular or neuropsychiatric-related disorders, as well as previous history of hospitalization, were at risk for long COVID [215,220,221].

The autoimmunity to the autonomic nervous system may explain the diversity of the clinical manifestations. In this regard, it has been proposed that the viral S protein could damage the endothelium in an animal model, disrupt the blood–brain barrier (BBB), and cross the BBB, resulting in perivascular inflammation [222,223]. These findings suggest an involvement of immune-related dysfunction in the development of post-COVID syndrome and have led to the proposal of immunomodulatory treatments in conjunction with vaccination against COVID-19 in patients with long-term COVID-19.

The impact of vaccination either before or after SARS-CoV-2 and its effect on long COVID has been extensively studied. While many of the symptoms mimic the ones seen during SAE, like fatigue, muscle pain, and chest pain, studies comparing unvaccinated and vaccinated people showed that vaccination reduces the risk of developing long COVID [224,225]. Interestingly, the protection was better with every additional dose of the COVID-19 vaccine and overall, at least after any one dose of vaccination [225,226,227,228]. The immunogenic effect following vaccination offers protection in reducing the symptoms of long COVID, thus being an advantage for all at-risk populations that may benefit.

In a cross-sectional study in the US, a comparison of booster vaccinations of COVID-19, primary vaccination, and incomplete vaccinations. The study showed that one booster dose reduced the odd ratio of long COVID by 24% and is better than primary vaccination alone, encouraging the booster vaccines for COVID-19 to be administered to prevent long COVID [229]. In a systematic review of 16 observational studies that included 614, 392 individuals studied the effect of mRNA vaccines (Pfizer-BioNTech and Moderna) as well as adenoviral vector vaccines (Ad26.COV2.S and ChAdOx1) on long COVID post-vaccination were compared. The study showed that one, two, and three or more COVID-19 vaccine doses reduced the risk of long COVID by 21, 59, and 73 percent, respectively [226]. In another systematic review and meta-analysis of 32 studies comprising 775,931 individuals, vaccination was effective against long COVID by 36.8 and 68.7 percent after two and three doses of COVID-19 vaccines [230].

## 10. Key Challenges in the Management of COVID-19 Vaccination Strategies and Recommendations

According to the WHO, COVID-19 vaccination helps to reduce the number of deaths due to the disease, particularly in immunocompetent individuals. However, crucial knowledge gaps exist in evaluating the same in a heterogeneous population with a spectrum of host immune responses. Some of the challenges are discussed below:

1. At least one dose of the COVID-19 vaccine has been recommended for people who have never received any vaccine but are at high risk of getting COVID-19, including old age/sick, immunocompromised people, and healthcare workers. This concept poses the question, “Should people who are already affected by COVID-19 need a vaccine?”. Studies comparing natural infection and hybrid immunity show that the latter immunity is superior to the former and unvaccinated individuals in terms of humoral and cell-mediated immunity. Therefore, integration of the history of COVID-19 before vaccination and the collection of more seroprevalence information on the conditions can help to devise effective immunization strategies.
2. At least one dose of COVID-19 vaccine is recommended for pregnant women. As discussed in this review, studies show that pregnant women are at higher risk of hospitalization due to COVID-19. Furthermore, since pregnant women, particularly ones with low education and low income, show vaccine hesitancy, they were encouraged to take the vaccine. However, vaccinated pregnant women have the same level of maternal and/or neonatal adverse events as compared to unvaccinated pregnant women. Thus, the benefit of vaccination in this population remains unclear and warrants more detailed clinical studies.
3. Compared to healthy individuals, those with a higher risk were recommended for revaccination after 6 to 12 months of the most recent dose. Previous studies that assessed the safety, immunogenicity, and reactogenicity with booster doses after the primary vaccination with one, two, three, or more doses showed better protection and fewer systemic side effects than those that received only the primary dose. Additionally, studies showed that homologous boosters had fewer systemic side effects than heterologous boosters. However, the impact of SARS-CoV-2 infection-induced immunity after the first or booster vaccination on potential side effects later remains unknown.
4. The WHO considers healthy infants/children aged 6 months to 17 years as low priority group for COVID-19 vaccination. In addition, vaccine hesitancy is observed among the parents of infants compared to school-going children. Nonetheless, studies showed no adverse events except for pain at the injection site, malaise, and fever for vaccinated children. However, the long-term effect of vaccination in this group, with or without COVID-19, remains unknown.
5. Since COVID-19 can cause anaphylaxis due to immediate-type hypersensitivity, and people with a previous history are at risk for anaphylaxis, vaccines are not recommended for this group of people with a history of severe allergy or anaphylaxis. Therefore, the guidelines for vaccination in this group need to be evaluated considering the long and short-term adverse effects.
6. Most of the adverse effects of vaccination were reported in the adult population of 18–64 years. However, studies showed female preponderance for some conditions like anaphylaxis, vaccine-induced immune thrombotic thrombocytopenia, and neurological dysfunction, including demyelinating diseases, Bell’s palsy, autoimmune encephalitis, small fiber neuropathy, and psychotic dysfunction. Importantly, the vaccine-induced adverse effects varied among adults and covered a wide range of ages and genders with no clear predisposition of the population to any one condition. Thus, more nuanced clinical studies are warranted to identify risk groups for these delayed-type adverse effects and if some populations can be selectively restrained from vaccination to reduce the same.
7. The immediate and delayed-type autoimmune reactions and inflammatory responses attributed to most of the adverse reactions associated with the COVID-19 vaccination. The molecular mechanisms include molecular mimicry, bystander activation, epitope spreading, antibody-dependent enhancement, and vaccine adjuvant-induced inflammatory effects. One or more mechanism(s) could lead to vaccine-induced adverse events in patients. However, the impact of vaccination before or after COVID-19 on the host immunity and adverse effects, both long and short-term, remains unknown. Therefore, there is a need to identify any risk factor that could trigger one or more mechanisms leading to autoimmune conditions among vaccinated individuals with or without COVID-19.

Following COVID-19, individuals develop “post or long-COVID syndrome” that typically starts at about 3 months post SARS-CoV-2 infection and persists for 2 months or longer, with varied symptoms and severity in multi-organ ailments. The management of this condition is currently symptom-based, and vaccination is suggested to help in overcoming the chronic post/long-COVID syndrome. However, more clinical data are needed to evaluate the beneficial effect of vaccination in preventing or reducing long-term COVID-19 in various population groups, including children, pregnant women, and immunocompromised individuals.

## 11. Summary and Conclusions

Worldwide, there are 775,754,322 cases of COVID-19, with 7,053,902 cases of death reported until 7 July 2024 from its beginning on 12 December 2019 [1]. Conventionally, vaccine development typically involves various stages of development where research identifies an antigen either by mathematical or bioinformatic approach dependent or independent of a wet lab activity. Preclinical studies involving cell-based and animal models would be conducted to evaluate immunogenicity, efficacy, and safety. Following this, clinical trials are conducted in various groups of populations comprising phases I, II, III, or even IV with specified periods before being licensed for use by approving bodies like the Food and Drug Administration (FDA) of the United States of America (USA), or the European Medicines Agency in European Union (Europe) [231,232]. In contrast to the conventional method, COVID-19 vaccines were developed in 12–18 months, which included parallel clinical trials, data from previous Coronavirus studies, and emergency use listing of candidates in phase III or IV before licensing [231,233].

Manufacturing of vaccines involves quality-related regulatory requirements, including the biological origin of antigens, adjuvants, and media components. Formulation of active ingredient, potency, storage temperature, and stability over time. In the case of a COVID-19 vaccine owing to a global emergency, a rolling review approach was offered that provided flexibility to review the vaccines and authorize them even before the complete dossier is ready [232,234,235]. Previous reports have shown that there are 183 vaccine candidates in clinical development, with eleven of them recommended in emergency use listing (EUL), namely Serum Institute of India (COVOVAX (Novavax formulation), Novavax (Nuvaxovid), Moderna (mRNA-1273), Pfizer/BioNTech (BNT162b2), CanSino (Convidecia), Janssen (Johnson & Johnson) (Ad26.COV2-S), Oxford/AstraZeneca (ChAdOx1-S), Serum Institute of India (ChAdOx1-S), Bharat Biotech (BBV152), Sinopharm (Covilo) and Sinovac (CoronaVac) [6]. Till June 2021, only four vaccines were listed under WHO EUL for COVID-19 vaccines, which include two mRNA vaccines—Moderna (mRNA-1273), Pfizer/BioNTech (BNT162b2) and two Adenoviral vector-based vaccines—Janssen (Johnson & Johnson) (Ad26.COV2-S), Oxford/AstraZeneca (ChAdOx1-S) [232]. So far, 5.47 billion COVID-19 vaccine doses have been administered as primary or booster doses, and by far, many countries have used these four vaccines predominantly [1].

The novelty of this review is that, for the first time, it comprehensively highlights the side effects in vaccinated but not infected individuals. Further, while few previous reviews have highlighted the acute side effects of a few COVID-19 vaccines, this review summarizes the chronic side effects of a broad range of COVID-19 vaccines, which is just emerging. Our literature search about the adverse impact identified predominantly two vaccine types and products aforementioned in the review. However, there could be incidents of adverse effects that are pertinent to specific risk groups or population groups. This is a limitation of the current report whereby adverse effects caused specifically by certain vaccines used in specific countries or beyond the emergency use listing could be missed. However, currently, there is not much information known about these special circumstances, and as/when more data emerge on these aspects, the future review(s) will be updated with such knowledge accordingly.

The WHO’s Strategic Advisory Group of Experts on Immunization has few recommendations for the COVID-19 vaccine among all populations—adults, adolescents, and children with co-morbidities who never received any prior COVID-19 vaccination to take one dose. For individuals with one previous dose of the COVID-19 vaccine, high priority is given to adults over 75–80 years of age and adults over 50–60 years old with co-morbidities to take a booster in 6–12 months of primary vaccination as well as adults over 50–60 years and adults with co-morbidities to take a booster 12 months after primary vaccine. People with a previous history of severe allergy/anaphylaxis are not recommended to take the vaccine [236].

With the current situation of the rapid necessity of COVID-19 vaccination combined with the EUL status of many vaccines, it is understood that the global pandemic situation has called for the necessity to use vaccines to save lives from severe forms of COVID-19 disease, including death. However, considering various facts of immune response to COVID-19 vaccines, most of which are varied and very specific in terms of organ system affected. Overall, adults in the age group of 18–64 years have displayed various adverse effects, which could be multifactorial considering the clinical condition, immune status, age, and gender of the person. Although adverse events are reported, it is rare compared to the number of vaccine doses given and the fact that COVID-19 deaths have reduced by 2023 in comparison to 2021 and 2022, when the vaccines were beginning to be given globally. Although the adverse effects are highlighted, an additional reason is the attention drawn to COVID-19 as compared to other pandemics and the visibility of all associated events. It is recommended by the CDC and WHO that the potential beneficial effect of the vaccine should outweigh the vaccination anxiety among adults and adolescents, though it is important to carefully monitor the adverse events.

## Figures and Tables

**Table 1:** Description of various types of COVID-19 vaccines.

Type of Vaccine	Antigen	Clinical Trials * (Phase 3/4)	Types of Administration	Dose Schedule (Predominant)	Advantages **	Disadvantages **
Inactivated	Whole virus	22 (13)	IM	Day 0 + 14 Day 0 + 21	Safe and easy to prepare; strong immune response due to native antigen expression; storage is easy.	Requires adjuvants; safety especially administration in immunocompromised patients.
Live attenuated	Whole virus	2 (1)	IN	Day 0	Stronger immune response; native antigen expression.	Requires adjuvants; safety especially administration in immunocompromised patients.
Protein subunit	S protein of SARS-CoV-2	59 (23)	IM	Day 0 + 21 or Day 0 + 28	Safe and well-tolerated; stable.	Moderate immunogenicity; requires adjuvants.
DNA vaccines	Plasmid DNA encoding the S protein of SARS-CoV-2	17 (2)	ID	Day 0 + 28 + 56	Good stability; safe to handle; relatively inexpensive to generate.	Moderate immunogenicity; requires adjuvants or nanoparticles; repeated doses; risk of integration into the host genome.
mRNA vaccines	S protein mRNA	43 (10)	IM, ID or SC	Day 0 + 14 Day 0 + 21 Day 0 + 28	Rapid research and development; simple production process; no risk of integration into the host DNA; strong immunogenicity.	mRNA is unstable and easily degraded; storage; requires adjuvants or nanoparticles; repeated doses.
Virus-like particle	Virus multimeric particles	7 (3)	IM	Day 0 + 21	VLPs are faster to manufacture; stable; no risk for use with immunocompromised individuals.	Not recommended for emergency use.
Non-replicating viral vector	Non-replicating viral vector carrying S protein of SARS-CoV-2	26 (7)	IM and IN	Day 0 Day 0 + 21 Day 0 + 56	Rapid research and development; strong immunogenicity; long-term protection.	Risk of pre-existing immunity against the viral vector used; cost.
Replicating viral vector	Replicating viral vector carrying S protein of SARS-CoV-2	6 (1)	IM	Day 0 + 28	Strong immunogenicity; long-term protection.	Risk of pre-existing immunity against the viral vector used; cost.

IM—intramuscular; IN—intranasal; SC—subcutaneous; ID—intradermal. * The safety and efficacy of different COVID-19 vaccine types listed here including details of clinical trials have been previously described [24,25,26,27]. ** The advantages and disadvantages of the COVID-19 vaccine types listed here have been described previously [29,30,31].
